# Clinical application of free-breathing 3D whole heart late gadolinium enhancement cardiovascular magnetic resonance with high isotropic spatial resolution using Compressed SENSE

**DOI:** 10.1186/s12968-020-00673-5

**Published:** 2020-12-17

**Authors:** Lenhard Pennig, Simon Lennartz, Anton Wagner, Marcel Sokolowski, Matej Gajzler, Svenja Ney, Kai Roman Laukamp, Thorsten Persigehl, Alexander Christian Bunck, David Maintz, Kilian Weiss, Claas Philip Naehle, Jonas Doerner

**Affiliations:** 1 Institute for Diagnostic and Interventional Radiology, Faculty of Medicine and University Hospital Cologne, University of Cologne, Kerpener Straße 62, 50937 Cologne, Germany; 2 Department of Radiology, Massachusetts General Hospital, Harvard Medical School, 55 Fruit Street, White 270, Boston, MA 02114 USA; 3 Else Kröner Forschungskolleg Clonal Evolution in Cancer, University Hospital Cologne, Weyertal 115b, 50931 Cologne, Germany; 4 Department III of Internal Medicine, Heart Center, Faculty of Medicine and University Hospital Cologne, University of Cologne, Kerpener Straße 62, 50937 Cologne, Germany; 5 Department of Radiology, University Hospitals Cleveland Medical Center, 11000 Euclid Ave, Cleveland, OH 44106 USA; 6 Department of Radiology, Case Western Reserve University, 11000 Euclid Ave, Cleveland, OH 44106 USA; 7 Philips GmbH, Röntgenstraße 22, 22335 Hamburg, Germany

**Keywords:** Late gadolinium enhancement, Cardiovascular magnetic resonance, Cardiomyopathy

## Abstract

**Background:**

Late gadolinium enhancement (LGE) cardiovascular magnetic resonance (CMR) represents the gold standard for assessment of myocardial viability. The purpose of this study was to investigate the clinical potential of Compressed SENSE (factor 5) accelerated free-breathing three-dimensional (3D) whole heart LGE with high isotropic spatial resolution (1.4 mm^3^ acquired voxel size) compared to standard breath-hold LGE imaging.

**Methods:**

This was a retrospective, single-center study of 70 consecutive patients (45.8 ± 18.1 years, 27 females; February–November 2019), who were referred for assessment of left ventricular myocardial viability and received free-breathing and breath-hold LGE sequences at 1.5 T in clinical routine. Two radiologists independently evaluated global and segmental LGE in terms of localization and transmural extent. Readers scored scans regarding image quality (IQ), artifacts, and diagnostic confidence (DC) using 5-point scales (1 non-diagnostic—5 excellent/none). Effects of heart rate and body mass index (BMI) on IQ, artifacts, and DC were evaluated with ordinal logistic regression analysis.

**Results:**

Global LGE (n = 33) was identical for both techniques. Using free-breathing LGE (average scan time: 04:33 ± 01:17 min), readers detected more hyperenhanced lesions (28.2% vs. 23.5%, P < .05) compared to breath-hold LGE (05:15 ± 01:23 min, P = .0104), pronounced at subepicardial localization and for 1–50% of transmural extent. For free-breathing LGE, readers graded scans with good/excellent IQ in 80.0%, with low-impact/no artifacts in 78.6%, and with good/high DC in 82.1% of cases. Elevated BMI was associated with increased artifacts (P = .0012) and decreased IQ (P = .0237). Increased heart rate negatively influenced artifacts (P = .0013) and DC (P = .0479) whereas IQ (P = .3025) was unimpaired.

**Conclusions:**

In a clinical setting, free-breathing Compressed SENSE accelerated 3D high isotropic spatial resolution whole heart LGE provides good to excellent image quality in 80% of scans independent of heart rate while enabling improved depiction of small and particularly non-ischemic hyperenhanced lesions in a shorter scan time than standard breath-hold LGE.

## Background

Cardiovascular magnetic resonance (CMR) late gadolinium enhancement (LGE) imaging has become the non-invasive standard of reference for assessment of myocardial viability in different cardiac diseases [1–3]. In ischemic cardiomyopathy, accurate assessment of the extent of infarct transmurality by LGE as a predictive indicator for prognosis and recovery of contractile function guides revascularisation therapy [4–7]. For non-ischemic cardiomyopathies, LGE enables differentiation among diseases based on different patterns of hyperenhancement [1, 8–10].

Traditionally, a two-dimensional (2D) inversion-recovery fast spoiled gradient-echo sequence is applied for LGE imaging with manually chosen inversion time (TI) to null the signal of healthy myocardium [1, 11]. However, 2D acquisition requires multiple breath-holds in pre-defined orientations with associated disadvantages such as non-isotropic resolution with thick slices, incomplete coverage due to slice gaps, low signal-to-noise ratio (SNR), and slice misregistration [11, 12].

The recent development of three-dimensional (3D) imaging allows data acquisition of the entire heart in a single scan without interslice gaps [3, 13]. In this context, accelerated and extended breath-hold approaches have been proposed [14, 15]. However, these techniques are associated with similar constraints on SNR and spatial resolution as 2D LGE since they can only provide highly anisotropic readouts [14, 15]. Further, they are hampered by long breath-hold duration, hence widely unsuitable for clinical routine [15, 16]. When applying different techniques for navigator-gating such as respiratory bellows signal [17], respiratory self-navigation [18–20], and diaphragmatic pencil-beam navigation [3] to reduce respiratory artifacts, 3D LGE can be performed during free-breathing enabling 3D assessment of the whole heart with higher spatial resolution [21–24]. Nevertheless, free-breathing 3D LGE techniques suffer from long imaging time, especially when aiming to provide readouts with submillimetre high isotropic spatial resolution [24–26]. Consequently, drawbacks like susceptibility to varying heart rate and respiratory motion as well as changing accumulation of contrast agent in injured myocardium with inadequate nulling of healthy tissue question their feasibility and usefulness in clinical routine [27, 28].

Recently, Compressed SENSE was introduced, which combines compressed sensing and parallel imaging using SENSitivity Encoding (SENSE) [29–31]. Compressed SENSE enables image-acquisition acceleration currently not achievable by compressed sensing or parallel imaging alone and has shown promising results in musculoskeletal and cardiovascular imaging [29–36].

The purpose of this study was to investigate the clinical potential of Compressed SENSE accelerated free-breathing 3D whole heart LGE with high isotropic spatial resolution compared to standard breath-hold LGE imaging for assessment of left ventricular (LV) viability.

## Methods

### Ethics

The institutional review board approved this single-center study (Ethikkommission, Medizinische Fakultät der Universität zu Köln; reference number: 19-1619). Given its retrospective design, written informed consent was waived for the patient cohort.

### Patient population

We retrospectively reviewed our internal database at our tertiary care medical center for CMR studies performed between February and November 2019. Patients were included if they received a standardized protocol in clinical routine for assessment of LV myocardial viability with known/suspected ischemic or non-ischemic cardiomyopathy including both, breath-hold and free-breathing LGE sequences. Patients were excluded in case of (I) inadequate nulling of breath-hold LGE or (II) uncertainty in assessment of global LGE in breath-hold sequences as determined by a board certified cardiovascular radiologist with eight years of experience in CMR (J.D.), and (III) a very low navigator efficiency of free-breathing LGE.

The following data were obtained from the medical charts or observed during examination: Patient age, gender, body mass index (BMI) at examination date, indication for viability study, heart rate during LGE imaging, LV ejection fraction (LVEF) as assessed by short-axis (SAx) cine, and scan time for LGE sequences. Final diagnosis of cardiomyopathies was based on multidisciplinary evaluation of clinical data, laboratory examination, biopsy results if available, echocardiographic findings, and CMR examination (breath-hold LGE) by the treating cardiology consultant and a board certified cardiovascular radiologist.

### Imaging

All CMR examinations were performed on a commercially available whole body 1.5T CMR system (Philips Ingenia, Philips Healthcare, Best, The Netherlands) equipped with a dedicated 28-channel coil for cardiac imaging, which consists of standard system-provided 12-channel posterior and 16-channel anterior coils. The clinical imaging protocol comprised 2D balanced steady-state free precession (bSSFP) breath-hold cine imaging in standard orientations (4-chamber (4Ch), 2-chamber (2Ch), 3-chamber (3Ch), and SAx) followed by admission of Gadobutrol (Gadovist, Bayer HealthCare Pharmaceuticals, Berlin, Germany; 0.2 mmol/kg), which was automatically injected into an antecubital vein at a flow-rate of 2 ml/s. Ten minutes after application of contrast agent, 2D gradient-echo T1-weighted sequence (Look-Locker) was used to determine TI adjusted to null LV myocardium.

#### Breath-hold LGE

As clinical standard, electrocardiogram (ECG)-triggered (end-diastolic) 3D Cartesian T1-weighted inversion-recovery fast spoiled gradient-echo multislice breath-hold LGE sequences covering the left ventricle in standard orientations (4Ch, SAx, 2Ch, and 3Ch) were acquired. The following imaging parameters were applied: Slice thickness 10 mm, shot duration 170 ms, repetition time 3.5 ms, echo time 1.7 ms, flip angle 15°, field of view (FOV) 300 × 239 × 50 mm^3^ (4Ch, SAx)/280 × 204 × 50 mm^3^ (3Ch, 2Ch), matrix 172 × 119 (4Ch, SAx)/176 × 133 (3Ch, 2Ch), acquired voxel size 1.64 × 1.72 × 10 mm^3^, and reconstructed voxel size 1.59 × 1.59 × 10 mm^3^. Spectral fat suppression was used to avoid high-intensity signals from epicardial fat. Scan time was 18 s per breath-hold; to cover the SAx, two stacks were acquired.

#### Free-breathing LGE

**Fig. 1:**
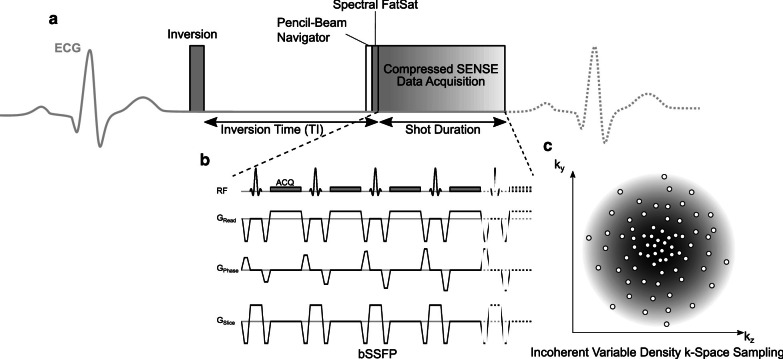
Schematic pulse sequence diagram of free-breathing late gadolinium enhancement (LGE). **a** The pulse sequence is electrocardiogram (ECG)-triggered to end-diastolic cardiac phase with TI based on the estimation for breath-hold LGE with the additional time delay of free-breathing LGE being taken into account by adding 50 ms. Prior to data acquisition, a pencil-beam navigator for respiratory gating to end-expiratory phase is employed followed by Spectral FatSat (spectral fat suppression) to avoid signal contribution by epicardial fat. **b** Imaging data is acquired using balanced steady-state free precession (bSSFP). In every cardiac cycle, 35 k-space lines are acquired, resulting in a shot duration of 150 ms. **c** A variable density incoherent sampling patter with high-density in the k-space center and continuously decreasing sampling density towards the k-space periphery was employed for data acquisition using Compressed SENSE

Free-breathing whole heart 3D Cartesian LGE was acquired immediately after breath-hold LGE. Figure 1 shows a schematic pulse sequence diagram of the free-breathing LGE sequence. To compensate for cardiac and respiratory motion, ECG-triggering (end-diastolic) in combination with diaphragmatic pencil-beam navigation was applied. The navigator was placed on the dome of the right hemidiaphragm and a 6 mm gating window during end-expiration was employed. TI as estimated for breath-hold LGE sequences was increased by 50 ms. Similar to breath-hold LGE, spectral fat suppression was employed. bSSFP was used for data acquisition as it allows for a very short repetition time, which is beneficial given the time constraints in high-resolution isotropic LGE imaging and since less signal is lost due to signal spoiling compared to gradient-echo imaging. Imaging parameters were as follows: Repetition time 4.4 ms, echo time 2.2 ms, flip angle 45°, FOV 298 × 265 × 120 mm^3^, matrix 188 × 212 × 171, acquired voxel size 1.40 × 1.40 × 1.40 mm^3^, and reconstructed voxel size 0.70 × 0.69 × 0.69 mm^3^. In every cardiac cycle, 35 k-space lines were acquired resulting in a shot length of 150 ms in end-diastolic cardiac phase.

For acceleration of image acquisition, Compressed SENSE (*Philips Healthcare *) was used. Data was acquired using a balanced variable density incoherent sampling patter within high-density in the k-space center and continuously decreasing sampling density towards the k-space periphery. For image reconstruction, an iterative L1 norm minimization ensuring data consistency and sparsity in the wavelet domain in combination with regularization by coil sensitivity distribution and SENSE parallel imaging was used. Image reconstruction, which was performed with standard hardware provided by the manufacturer of the CMR system, took about 30 s. An acceleration factor of 5 was employed, resulting in a nominal scan time of 02:08 min or 04:16 min when considering 50% of navigator efficiency, and a heart rate of 60 beats per minute. Data were acquired in the transaxial plane.

### Image analysis

For multiplanar reformatting of free-breathing LGE source images, a radiologist with four years of experience in CMR (M.S.) used the Multiplanar-Reconstruction-(MPR) tool in a commercially available image viewer (IMPAX EE, release 20; Agfa HealthCare N.V., Mortsel, Belgium) to create reconstructions in standard orientations (4Ch, SAx, 2Ch, and 3Ch) with identical slice thickness as breath-hold LGE scans. Scans were than evaluated by two radiologists with four (A.W., R1) and three (L.P., R2) years of experience in CMR using the same IMPAX EE (release 20, Agfa HealthCare N.V) workstation during separate reading sessions. Readers were blinded to clinical and patient data. The reading took place in two sessions: In the first session, the readers evaluated breath-hold LGE scans whereas in the second session, they assessed reconstructed free-breathing LGE images and its source images with the latter being free to visualize in all three directions of space using the MPR-tool. A period of four weeks between sessions was used to minimize recall bias. Readers were free to adjust window leveling.

#### Assessment of LGE

Readers assessed scans for global and segmental LGE of the LV applying the American Heart Association’s 17-segment model [37]. Hyperenhanced lesions were scored for each segment based on the area of scar per segment (0 = no hyperenhancement, 1 = 1–25%, 2 = 26–50%, 3 = 51–75%, and 4 = 76–100% hyperenhancement) and on the location of scar within the segment (1 = subendocardial, 2 = mid-myocardial, 3 = subepicardial, and 4 = transmural). Further, readers analyzed scans for potential pericardial hyperenhancement (0 = no hyperenhancement, 1 = left ventricular, 2 = right ventricular, and 3 circumferential) and intracardiac thrombi.

#### Assessment of image quality, artifacts, and diagnostic confidence

Using 5-point scales, readers scored LGE sequences in terms of image quality (1: non-diagnostic, image quality inadequate for diagnosis; 2: poor, suboptimal image quality for diagnosis; 3: fair, mediocre image quality acceptable for diagnosis; 4: good, image quality suitable for confident diagnosis; and 5: excellent, image quality providing highly confident diagnosis) and artifacts considering blurring artifacts, banding artifacts, respiratory artifacts, cardiac motion artifacts, and parallel imaging reconstruction artifacts (1: non-diagnostic; 2: high impact, 3: moderate impact, 4: low impact, and 5: none) as well as diagnostic confidence (1: non-diagnostic, 2: low, 3: fair, 4: good, and 5: high).

### Statistical analysis

Statistical analysis was performed using JMP (release 14.1.0, SAS Institute, Cary, North Carolina, USA) with statistical significance being set to P < 0.05. Data are expressed as mean ± standard deviation, unless noted otherwise. Comparison of paired nonparametric variables and scan times was performed with Wilcoxon signed-rank tests. To compare the proportion of detected hyperenhanced lesions between LGE techniques, the McNemar test was applied. Cohen’s Kappa was used to evaluate interobserver agreement of detection of hyperenhanced lesions using free-breathing and breath-hold LGE sequences. The interpretation of an agreement was as follows: 0.01–0.2 slight, 0.21–0.4 fair, 0.41–0.6 moderate, 0.61–0.8 substantial, and 0.81–0.99 almost perfect agreement [38]. Influence of confounders (heart rate and BMI) on subjective evaluation of image quality, artifacts, and diagnostic confidence of LGE sequences was assessed using ordinal logistic regression analysis.

## Results

### Study population and baseline characteristics

Seventy-nine patients met the inclusion criteria. Thereof, four patients were excluded given inadequate nulling of breath-hold LGE, three due to uncertainty in assessment of global LGE in breath-hold sequences, and two given very low navigator efficiency of free-breathing LGE.

Consequently, 70 patients (27 females) were included in this study, yielding an age of 45.8 ± 18.1 years (range 16–80 years) and BMI of 25.1 ± 4.2 (17.6–37.4). The heart rate was 70 ± 12 (48–109) beats per minute and a LVEF of 60 ± 17% (19%–85%) was observed. Scans were acquired for viability assessment of known/suspected ischemic cardiomyopathy in 19 (27.1%) and non-ischemic cardiomyopathies in 51 (72.9%) patients. Thereof, 18 (35.3%) patients were examined for evaluation of potential (peri-)myocarditis, nine (17.6%) of structural heart disease, seven (13.7%) of sarcoidosis, six (11.8%) of dilated cardiomyopathy (DCM), five (9.8%) of hypertrophic cardiomyopathy (HCM), three (5.8%) of amyloidosis, two (3.9%) of chemotherapy-induced cardiomyopathy, and one patient (2.0%) of Anderson-Fabry disease.

### Imaging

All included imaging studies were executed successfully. Depending on patient’s heart rate and breathing frequency, free-breathing LGE took 04:33 ± 01:17 min to acquire (46.9% navigator efficiency). Breath-hold LGE sequences lasted 05:15 ± 01:23 min (P = .0104).

### Image analysis

#### Assessment of LGE

Global LV myocardial LGE (n = 33) was identical for both techniques and readers, present in 13 patients (39.4%) with ischemic and 20 patients (60.6%) with non-ischemic cardiomyopathy (P = 1.0). Thereof, findings consistent with (peri-) myocarditis were present in 11 (55.0%) patients, sarcoidosis in three (15.0%), DCM in two (10.0%), HCM in two (10.0%), amyloidosis in one (5.0%), and chemotherapy-induced cardiomyopathy in one (5.0%) patient.

Table 1 provides an overview of indications for viability studies and final LGE findings.

**Table 1:** Indications for viability studies and final late gadolinium enhancement (LGE) findings.

Indication for viability study	n = 70	Final LGE findings	n = 33
Ischemic cardiomyopathy	19 (27.1%)	Ischemic cardiomyopathy	13 (39.4%)
Non-ischemic cardiomyopathies	51 (72.9%)	Non-ischemic cardiomyopathies	20 (60.6%)
(Peri-) Myocarditis	18 (35.3%)	(Peri-) Myocarditis	11 (55.0%)
Structural heart disease	9 (17.6%)	Sarcoidosis	3 (15.0%)
Sarcoidosis	7 (13.7%)	Dilated cardiomyopathy	2 (10.0%)
Dilated cardiomyopathy	6 (11.8%)	Hypertrophic cardiomyopathy	2 (10.0%)
Hypertrophic cardiomyopathy	5 (9.8%)	Amyloidosis	1 (5.0%)
Amyloidosis	3 (5.8%)	Chemotherapy-induced cardiomyopathy	1 (5.0%)
Chemotherapy-induced cardiomyopathy	2 (3.9%)		
Anderson-Fabry disease	1 (2.0%)		

Individual and averaged segmental LGE assessment of readers is given in Table 2. Readers detected 132 hyperenhanced lesions in breath-hold (23.5%; R1: 126 (22.5%), R2: 138 (24.6%)) sequences whereas 158 lesions were noted using free-breathing LGE (28.2%; R1: 153 (27.3%), R2: 163 (29.1%); P < 0.0001).

**Table 2:** Left ventricular LGE assessment in patients with global LGE (n = 33) of both readers individually and averaged for both readers based on the 17-segment model in terms of localization and transmural extent

READER 1				
Overall detected hyperenhanced lesions in patients showing global LGE (n = 33)				
Breath-hold	n = 126/561 (22.5%) detected hyperenhanced lesions			P < .0001
Free-breathing	n = 153/561 (27.3%) detected hyperenhanced lesions			
LGE localization	*Subendocardial*	*Mid-myocardial*	*Subepicardial*	*Transmural*
Breath-hold	24/126 (19.1%)	25/126 (19.8%)	21/126 (16.7%)	56/126 (44.4%)
Free-breathing	31/153 (20.3%)	29/153 (19.0%)	32/153 (20.9%)	61/153 (39.9%)
LGE transmural extent	*1–25%*	*26–50%*	*51–75%*	*76–100%*
Breath-hold	16/126 (12.7%)	25/126 (19.8%)	25/126 (19.8%)	60/126 (47.6%)
Free-breathing	29/153 (18.9%)	32/153 (20.9%)	27/153 (17.6%)	65/153 (42.5%)
READER 2				
Overall detected hyperenhanced lesions in patients showing global LGE (n = 33)				
Breath-hold	n = 138/561 (24.6%) detected hyperenhanced lesions			P < .0001
Free-breathing	n = 163/561 (29.1%) detected hyperenhanced lesions			
LGE localization	*Subendocardial*	*Mid-myocardial*	*Subepicardial*	*Transmural*
Breath-hold	33/138 (23.9%)	24/138 (17.4%)	27/138 (19.6%)	54/138 (39.1%)
Free-breathing	39/163 (23.9%)	31/163 (19.0%)	38/163 (23.3%)	55/163 (33.7%)
LGE transmural extent	*1–25%*	*26–50%*	*51–75%*	*76–100%*
Breath-hold	23/138 (16.7%)	28/138 (20.3%)	31/138 (22.5%)	56/138 (40.6%)
Free-breathing	35/163 (21.5%)	38/163 (23.3%)	31/163 (19.0%)	59/163 (36.2%)
AVERAGED for both readers				
Overall detected hyperenhanced lesions in patients showing global LGE (n = 33)				
Breath-hold	n = 132/561 (23.5%) detected hyperenhanced lesions			P < .0001
Free-breathing	n = 158/561 (28.2%) detected hyperenhanced lesions			
LGE localization	*Subendocardial*	*Mid-myocardial*	*Subepicardial*	*Transmural*
Breath-hold	28.5/132 (21.6%)	24.5/132 (18.6%)	24/132 (18.2%)	55/132 (41.7%)
Free-breathing	35/158 (22.2%)	30/158 (19.0%)	35/158 (22.2%)	58/158 (36.7%)
LGE transmural extent	*1–25%*	*26–50%*	*51–75%*	*76–100%*
Breath-hold	19.5/132 (14.8%)	26.5/132 (20.1%)	28/132 (21.2%)	58/132 (43.9%)
Free-breathing	32/158 (20.2%)	35/158 (22.2%)	29/158 (18.4%)	62/158 (39.2%)

**Fig. 2:**
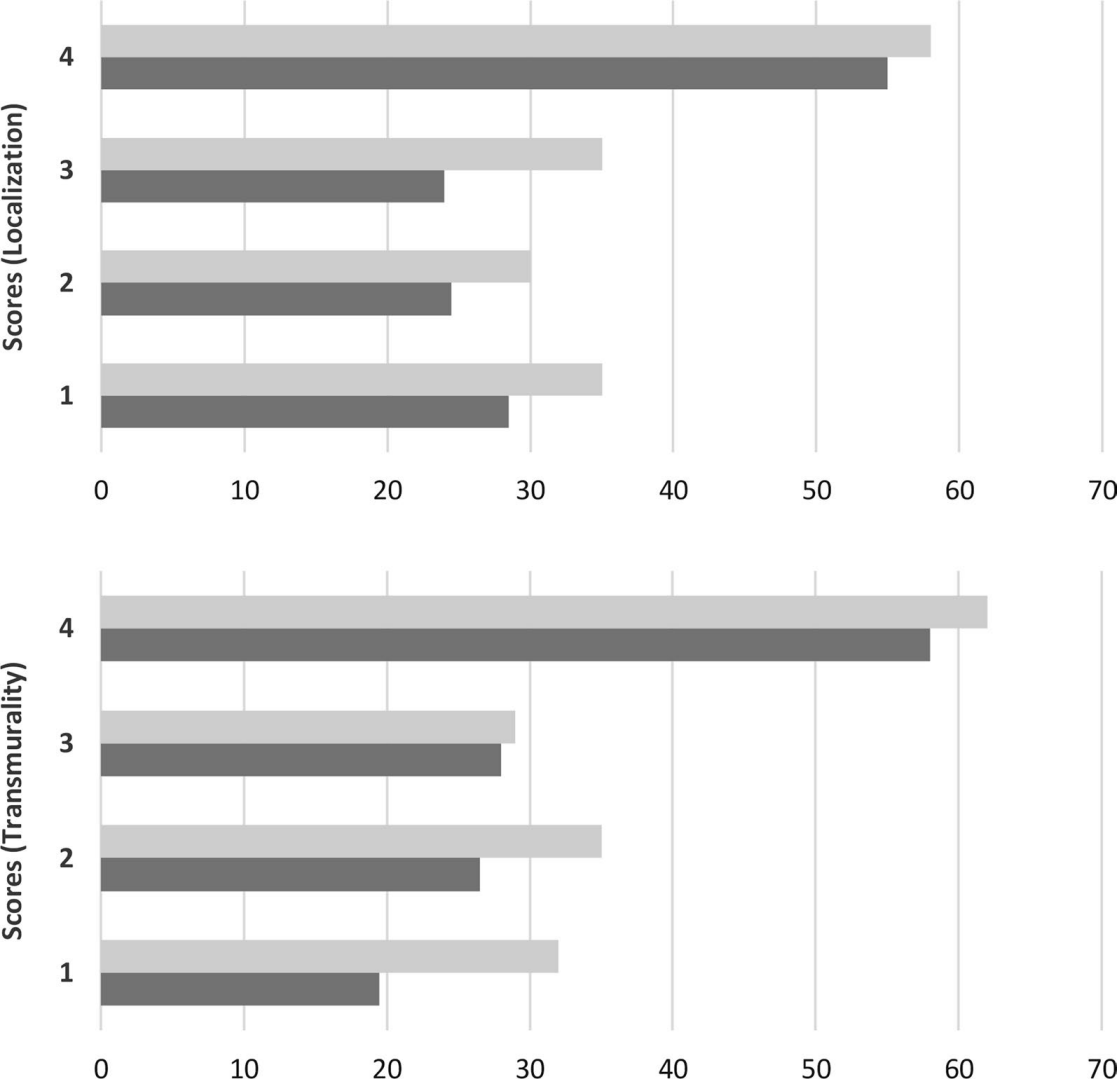
Distribution of hyperenhanced lesions in terms of localization within segment (1 = subendocardial, 2 = mid myocardial, 3 = subepicardial, 4 = transmural) and transmural extent (1 = 1–25%, 2 = 26–50%, 3 = 51–75%, 4 = 76–100% of the corresponding segment) for free-breathing LGE (light grey) and breath-hold LGE (dark grey) averaged for both readers

Averaged for both readers, depiction of transmural scar was comparable between both techniques (free-breathing: 58, breath-hold: 55) whereas readers identified more lesions in mid-myocardial (free-breathing: 30, breath-hold: 24.5), subendocardial (free-breathing: 35, breath-hold: 28.5), and subepicardial localization (free-breathing: 35, breath-hold: 24) in free-breathing LGE. Further, more small lesions were depicted using free-breathing LGE than with breath-hold LGE (1–25%: 32 vs. 19.5, 26–50%: 35 vs. 26.5) while identification of scars of 51–75% (free-breathing: 29, breath-hold: 28) and of 76–100% (free-breathing: 62, breath-hold: 58) of transmural extent was comparable between techniques. Figure 2 depicts distribution of lesions in terms of localization and transmural extent for free-breathing and breath-hold LGE.

For both techniques, an almost perfect interobserver agreement for detection of hyperenhanced lesions was observed [free-breathing LGE: Cohen’s Kappa of 0.94 (0.91–0.97), breath-hold LGE: Cohen’s Kappa of 0.93 (0.89–0.96)].

Concomitant pericardial LGE was identical for both techniques and readers (n = 9) with six patients presenting circular (66.7%), two LV (22.2%), and one patient right ventricular (11.1%) hyperenhancement (P = 1). Both readers detected intracardiac thrombi in three patients in both techniques (P = 1).

#### Assessment of image quality, artifacts, and diagnostic confidence

For both readers, free-breathing LGE achieved a score of 4.1 ± 0.8 for image quality, of 4.0 ± 0.9 for artifacts, and of 4.4 ± 0.8 for diagnostic confidence. Breath-hold LGE yielded scores of 4.4 ± 0.8 for image quality (P = .0028), of 4.3 ± 0.9 for artifacts (P = .0098), and of 4.5 ± 0.8 for diagnostic confidence (P = .3125).

Considering the overall distribution of scores of both readers combined, Table 3 gives detailed results. Breath-hold LGE provided good/excellent image quality in 87.1%, low-impact/no artifacts in 81.4%, and good/high diagnostic confidence in 90.7% of examinations. For free-breathing LGE, readers graded scans with good/excellent image quality in 80.0%, with low-impact/no artifacts in 78.6%, and with good/high diagnostic confidence in 82.1% of cases.

**Table 3:** Distribution of scores for image quality, artifacts, and diagnostic confidence of both readers combined for breath-hold and free-breathing LGE in absolute and relative values

Criterion	Technique	Non-diagnostic	Poor	Fair	Good	Excellent	Good + excellent
Image	Breath-hold	–	7 (5.0%)	11 (7.9%)	45 (32.1%)	77 (55.0%)	122 (87.1%)
quality	Free-breathing	–	7 (5.0%)	21 (15.0%)	61 (43.6%)	51 (36.4%)	112 (80.0%)

Criterion	Technique	Non-diagnostic	High impact	Moderate impact	Low impact	None	Low impact + none
Artifacts	Breath-hold	–	5 (3.6%)	21 (15.0%)	42 (30.0%)	72 (51.4%)	114 (81.4%)
	Free-breathing	–	11 (7.9%)	19 (13.6%)	63 (45.0%)	47 (33.6%)	110 (78.6%)

Criterion	Technique	Non-diagnostic	Low	Fair	Good	High	Good + high
Diagnostic	Breath-hold	–	6 (4.3%)	7 (5.0%)	40 (28.6%)	87 (62.1%)	127 (90.7%)
confidence	Free-breathing	–	4 (2.9%)	21 (15.0%)	33 (23.6%)	82 (58.6%)	115 (82.1%)

Table 4 displays influence of confounders (heart rate and body mass index) on subjective assessment of breath-hold and free-breathing LGE assessed by ordinal logistic regression analysis. Regarding breath-hold LGE, increased heart rate negatively influenced image quality (P = .0362) and artifacts (P = .0453). BMI did not yield significant influence on subjective assessment of breath-hold sequences. For free-breathing LGE, elevated BMI was associated with increased artifacts (P = .0012) and reduced image quality (P = .0237). Increased heart rate negatively influenced artifacts (P = .0013) and diagnostic confidence (P = .0479) whereas image quality (P = .3025) was unimpaired.

**Table 4:** Ordinal logistic regression analysis of influence of confounders on subjective assessment of breath-hold and free-breathing LGE expressed in P values, italics indicates statistical significance

Confounder	Criterion	Breath-hold	Free-breathing
Heart rate	Image quality	*.0362*	.3025
	Artifacts	*.0453*	*.0013*
	Diagnostic confidence	.1722	*.0479*
Body mass index	Image quality	.2520	*.0237*
	Artifacts	.8481	*.0012*
	Diagnostic confidence	.1822	.3523

**Fig. 3:**
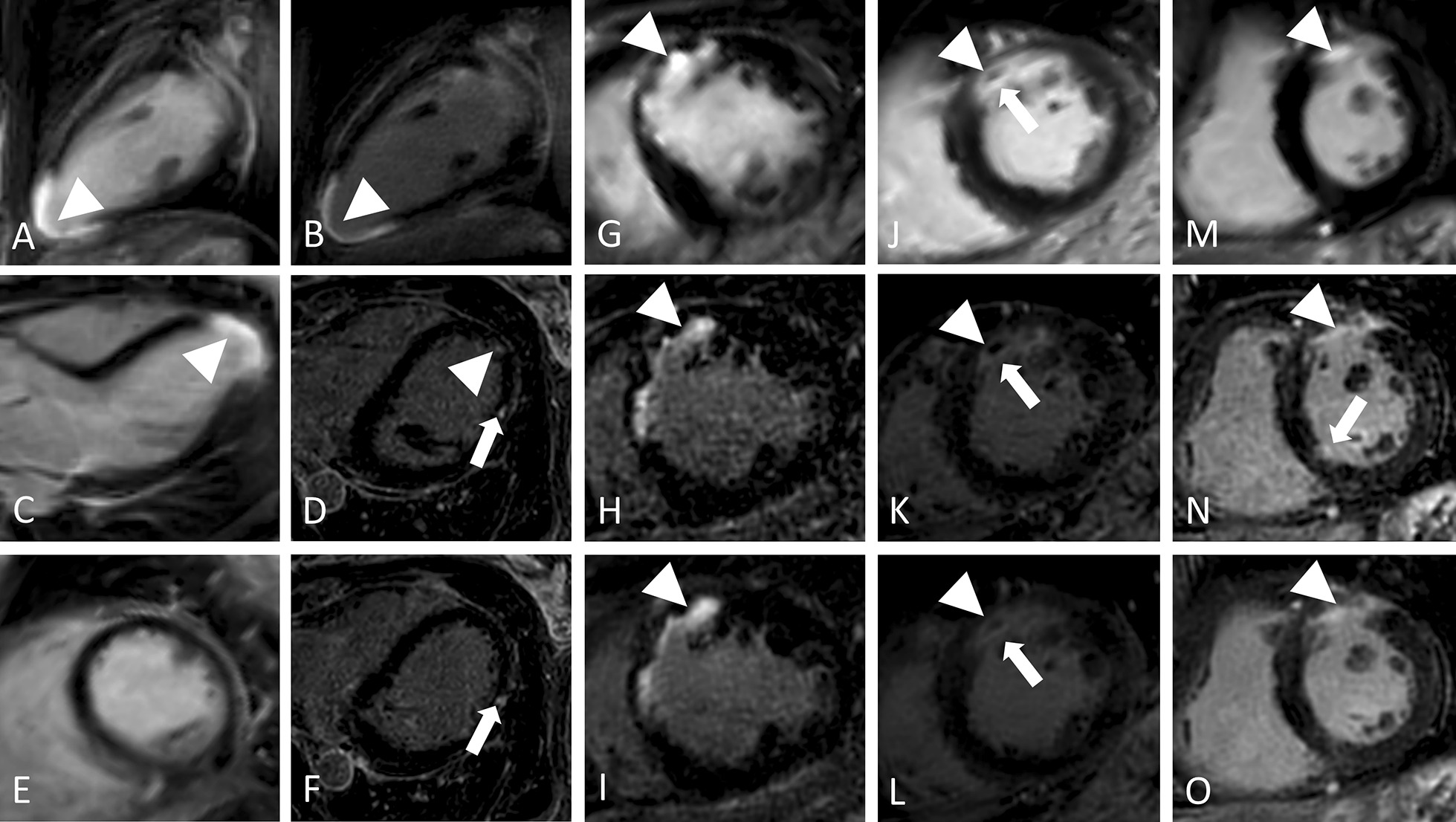
Performance of free-breathing LGE in ischemic cardiomyopathy in four different patients. 1. Seventy-year-old female with apical infarction (arrowheads) in breath-hold LGE in standard orientations (**a**, **c**, **e**) and reformatted two chamber (2Ch) (**b**) view of free-breathing LGE with identical slice thickness (10 mm) showing improved scar edge sharpness of the latter. Source images of free-breathing LGE (as acquired in transaxial plane; 0.7 mm slice thickness) reveal additional scattered hyperenhanced lesions (thin arrows) in mid-myocardial (**d**) and subepicardial (**f**) location at mid-ventricular level not visible in breath-hold LGE. 2. Forty-year-old female with anterior myocardial infarction (arrowheads) in short axis (SAx) (**g**, **h**, **i**) views. Compared to breath-hold LGE (**g**), reformatted free-breathing LGE (**h**: source images with 0.7 mm slice thickness, **i**: identical (10 mm) slice thickness as breath-hold LGE) enables improved depiction of subendocardial enhancement and assessment of transmural extent given its higher resolution and increased differentiation between blood and injured myocardium. 3. Seventy-four-year-old male with anterior myocardial infarction (arrowheads) as displayed in SAx (**j**, **k**, **l**) views. In comparison to breath-hold LGE (**j**), reformatted free-breathing LGE (**k**: source images with 0.7 mm slice thickness, **l**: identical (10 mm) slice thickness as breath-hold LGE) provides improved scar sharpness and facilitates delineation of microvascular obstruction (arrows). 4. Seventy-three-year-old male with anterior myocardial infarction (arrowheads) in SAx (**m**,** n**,** o**) views. Compared to breath-hold LGE (**m**), reformatted free-breathing LGE (**n**: source images with 0.7 mm slice thickness, **o**: identical (10 mm) slice thickness as breath-hold LGE) provides improved delineation of scar extent. Further, source images of free-breathing LGE (**n**) reveal an additional microinfarction basal inferoseptal (arrow) not visible in breath-hold LGE (**m**) or free-breathing LGE with identical slice thickness as breath-hold LGE (**o**).

**Fig. 4:**
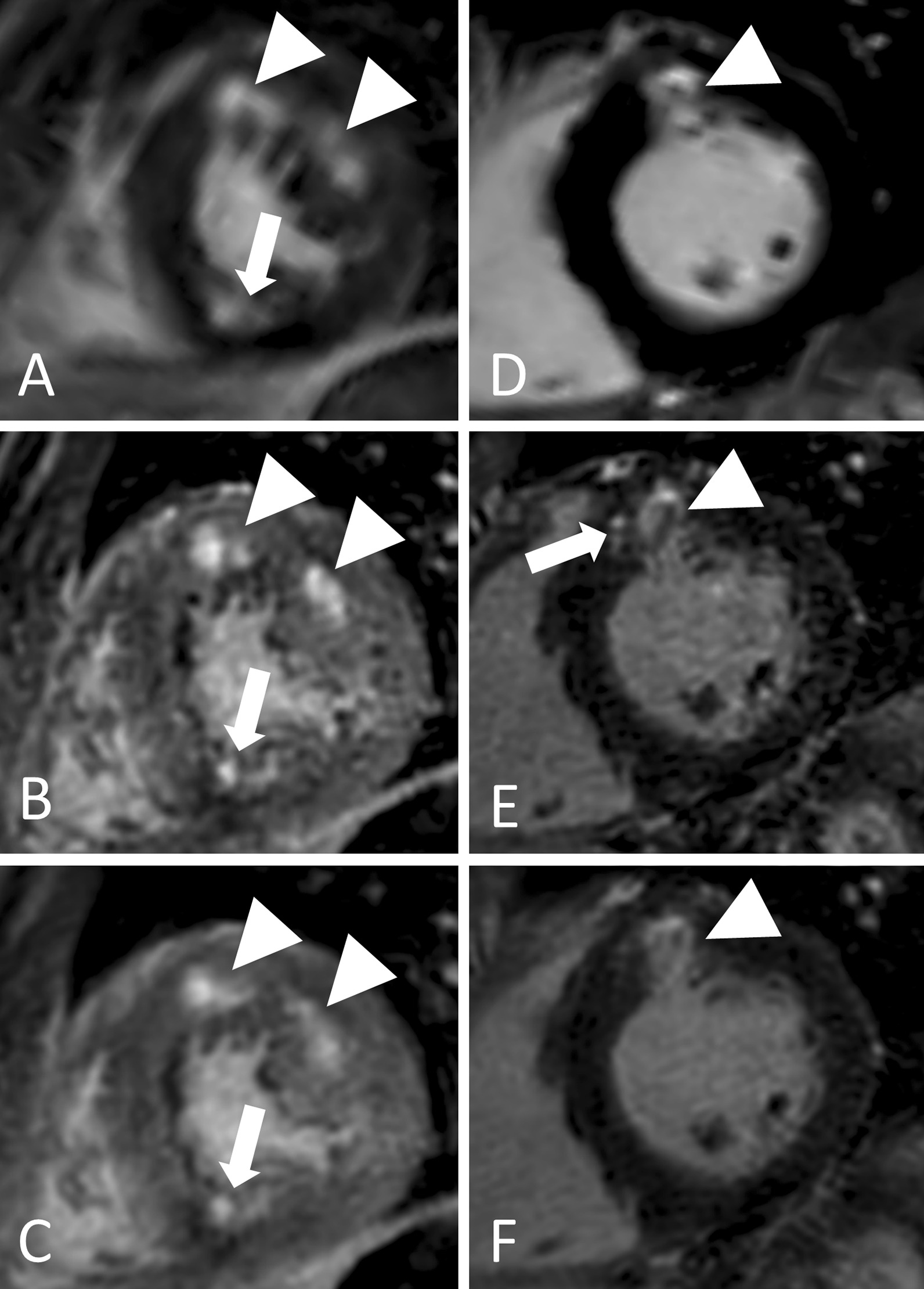
Performance of free-breathing LGE in non-ischemic cardiomyopathies in two different patients. 1. Twenty-year-old female with hypertrophic cardiomyopathy and patchy LGE in SAx views (**a**,** b**,** c**). Whereas in breath-hold LGE (**a**), hyperenhanced lesions at mid-anterior and mid-anterolateral segments (arrowheads) seem to be confluent, they can be distinguished as two separate lesions in reformatted free-breathing LGE (**b**: source images with 0.7 mm slice thickness, **c**: identical (10 mm) slice thickness as breath-hold LGE) despite suboptimal TI, which leads to a grey myocardium. Further, free-breathing LGE provides improved depiction of a subendocardial lesion at mid-inferior segment (arrows). 2. Fifty-year-old male with sarcoidosis and diffuse hyperenhancement of the anterior wall (arrowheads) in SAx views (**d**,** e**,** f**), pronounced at mid-myocardial localization. In its reformatted source images (**e**, 0.7 mm slice thickness), free-breathing LGE clearly depicts additional adjacent hyperenhanced lesions (arrow) of the mid-anteroseptal segment at mid-myocardial localization, which are indicated in free-breathing LGE with 10 mm slice thickness (**f**), but not visible in breath-hold LGE (**d**)

Exemplary comparisons of free-breathing and breath-hold LGE are given in Figs. 3 and 4.

## Discussion

In this study, we investigated the clinical potential of Compressed SENSE accelerated free-breathing 3D whole heart LGE with high isotropic spatial resolution compared to standard breath-hold LGE for assessment of LV viability. The major findings of the study are the following: 1. Free-breathing LGE enabled improved depiction of small hyperenhanced lesions of the LV myocardium, in particular of non-ischemic localization, and required a shorter scan time compared to breath-hold LGE. 2. Free-breathing LGE provided good to excellent results for diagnostic confidence, artifacts, and image quality in about 80% of examinations with the latter being independent of heart rate.

Given aforementioned limitations of breath-hold LGE, free-breathing LGE techniques are of particular interest in research and clinical practice. Previous studies have proposed different free-breathing approaches enabling high spatial resolution at 1.5 T and 3 T [18, 21–23, 25, 26]. However, long acquisition time remains one of the main limitations for the majority of techniques. In this context, Andreu et al. reported scan times of 16.4 ± 7.2 min (for 1.4 mm^3^ reconstructed resolution) [25], Bizino et al. of 09:34 ± 03:04 min (0.9 mm^3^ reconstructed resolution) [26], and Lintingre et al. of 8–10 min (1.25 × 1.25 × 2.5 mm acquired resolution) [24]. Bratis et al. introduced respiratory self-navigation for whole heart LGE in 03:52 min with 2.0 mm^3^ acquired and 1.0 mm^3^ reconstructed resolution [18] whereas Kino et al. demonstrated the use of phase-sensitive inversion-recovery (PSIR) for free-breathing LGE with 2.0 mm^3^ acquired resolution in 06:10 ± 02:56 min [23]. Akcakaya et al. [21] and Basha et al. [22], respectively, proposed another promising technique for free-breathing LGE providing the highest isotropic acquisition to date (varying between 1.0 and 1.7 mm^3^ depending on patient’s size and heart rate) in approximately 4 to 6 min (depending on applied acceleration factor) using LOw-dimensional-structure Self-learning and Thresholding (LOST). However, required reconstruction of acquired data using an offline central processing unit cluster, lasting about an hour, limits its immediate application in clinical routine and feasibility for other centers [21, 22].

In the present study, proposed Compressed SENSE accelerated free-breathing 3D whole heart LGE sequence enables high isotropic resolution in acquired (fixed 1.4 mm^3^, no adaptation to patient’s specifics required) and reconstructed (0.7 mm^3^) voxel size in an acceptable scan time (04:33 ± 01:17 min) without extensive postprocessing. Image reconstruction took about 30 s and was performed with standard hardware provided by the manufacturer of the CMR system.

As previously shown when comparing free-breathing to breath-hold LGE, readers detected more smaller hyperenhanced lesions using free-breathing LGE, mostly because of its contiguous, high-resolution acquisition in through-plane direction and because breath-hold LGE suffers from partial volume effect due to its lower voxel size [22–24, 39]. Although a voxel partially contains spins with high signal intensity, an overall elimination of high signal intensity of the voxel may occur if it is also occupied by spins with low signal intensity [39]. Therefore, small hyperenhanced lesions are more difficult to detect and to distinguish from another as two separate findings in breath-hold LGE. In breath-hold sequences, the partial volume effect may also lead to impaired detection of patchy mid-myocardial and, in particular, subepicardial lesions. In contrast, higher resolution of free-breathing LGE with possible 3D assessment facilitates their detection since it enables sufficient differentiation from adjoining structures.

Visually, the signal appearance of free-breathing LGE was lower compared to breath-hold LGE, which might have different reasons except for the distinct sequence details with free-breathing LGE being acquired at a later time point after administration of contrast agent and data acquisition comprising a comparable large timeframe. However, no quantitative SNR comparison was carried out in the current work because of the iterative and wavelet denoising based reconstruction used for free-breathing LGE, which is dependent on the actual image content and results in different numbers of iterations or denoising steps for different images interfering with absolute SNR estimations. The visually reduced signal of the blood pool improved detection of subendocardial lesions in this study, mostly due to visually moderately increased differentiation between blood and injured myocardium, facilitating depiction of scars at this localization, comparable to dark-blood LGE [40, 41].

Free-breathing LGE enabled sufficient detection of intracardiac thrombi and pericardial hyperenhancement. Given the low number of patients in the present study presenting respective pathologies, these findings may carry a bias and should warrant future investigations, including assessment of atrial and right ventricular LGE.

Free-breathing LGE did not fully yield the same high scores for image quality, artifacts, and diagnostic confidence as breath-hold LGE. These differences may be due to acquiring the free-breathing sequence at the end of examination when potential contrast washout leads to a change in optimal TI and patients tend to be exhausted and may present irregular breathing patterns. However, there was no statistical difference regarding diagnostic confidence between both techniques and free-breathing LGE achieved good/excellent image quality in 80% of cases. An earlier acquisition of free-breathing LGE may lead to an improvement of image quality and reduction of artifacts. Further, application of PSIR might reduce effects of dynamic changes of TI during examination [19, 23].

In free-breathing LGE, elevated BMI led to impaired image quality and more pronounced artifacts, while in breath-hold LGE, BMI had no impact on image quality and artifacts. It is of note, however, that diagnostic confidence of free-breathing was unimpaired by BMI. A possible explanation may be that the loss in image quality is counterbalanced by the improved post-processing options for free-breathing LGE such as multiplanar reformation. Further, increased heart rate led to decreased diagnostic confidence and increased artifacts for free-breathing LGE and affected artifacts as well as image quality of breath-hold LGE. However, increased heart rate did not lead to impaired image quality for free-breathing LGE. These findings differ to the study by Basha et al. investigating free-breathing LGE using LOST, for which increased heart rate was associated with significant decreased image quality [22]. The shorter shot duration of free-breathing LGE compared to breath-hold LGE (150 vs. 170 ms) may explain the findings of the present study.

Besides being acquired without any acceleration technique, the longer scan time of breath-hold LGE in this study is mostly due to patient recovery time between breath-holds and repetition of scans if the patient was unable to perform the breath-hold or if planning was not accurately. In contrast, free-breathing LGE can be performed in patients with poor compliance unable to tolerate repetitive breath-holds, proves to be widely user-independent, and enables post-acquisition reformatting in any arbitrary orientation with identical image quality as original transaxial images using multiplanar tools.

### Limitations

The following limitations need to be considered: First, there is no standard of reference for detection of LGE. In this study, breath-hold LGE was used for comparison since this technique is standard in clinical routine for assessment of LV viability. Histopathological staining or repeated acquisition of the same patient at the following day could have been conducted to confirm additional findings of free-breathing LGE but where not possible or desired given the retrospective setting and potential peri-interventional complications. Second, given the distinct appearance of LGE techniques, readers were not blinded to the type of sequence potentially influencing the results. Third, no direct comparison to other free-breathing (an) isotropic LGE techniques with different undersampling approaches was performed in the current study, which could nurture additional investigations. Finally, the possible impact of proposed free-breathing technique on final diagnosis of cardiomyopathies and further clinical decision making should be investigated in a prospective, multi-center setting, in which the order of acquisition of free-breathing and breath-hold LGE sequences is randomized to provide an unbiased evaluation.

## Conclusions

Free-breathing Compressed SENSE accelerated 3D whole heart LGE with high isotropic spatial resolution provides good to excellent image quality in 80% of scans independent of heart rate while enabling improved depiction of small hyperenhanced lesions with in particular non-ischemic localization of the LV myocardium in a shorter scan time than standard breath-hold LGE. Given its fast acquisition, wider user-independence, and omission of breath‐holds, Compressed SENSE accelerated free-breathing LGE has the potential to facilitate clinical workflow.

## Data Availability

The datasets generated and/or analyzed during the current study are not publicly available due to data protection but are available from the corresponding author upon reasonable request. The imaging protocol of proposed free-breathing LGE sequence is available from the corresponding author upon reasonable request.

## Abbreviations

2D: Two-dimensional
2Ch: 2-Chamber
3Ch: 3-Chamber
3D: Three-dimensional
4CH: 4-Chamber
BMI: Body mass index
bSSFP: Balanced steady-state free precession
CMR: Cardiovascular magnetic resonance
DCM: Dilated cardiomyopathy
ECG: Electrocardiogram
FOV: Field of view
HCM: Hypertrophic cardiomyopathy
LGE: Late gadolinium enhancement
LOST: LOw-dimensional-structure Self-learning and Thresholding (LOST)
LV: Left ventricle/left ventricular
LVEF: Left ventricular ejection fraction
MPR: Multiplanar-reconstruction
PSIR: Phase-sensitive inversion-recovery
SAx: Short-axis
SENSE: SENSitivity Encoding
SNR: Signal-to-noise ratio
TI: Inversion time
